# Microbial Production Potential of *Pantoea ananatis*: From Amino Acids to Secondary Metabolites

**DOI:** 10.3390/microorganisms10061133

**Published:** 2022-05-31

**Authors:** Yoshihiro Usuda, Yousuke Nishio, Gen Nonaka, Yoshihiko Hara

**Affiliations:** 1 Research and Business Planning Department, Ajinomoto Co., Inc., Tokyo 104-8315, Japan; 2 Research Institute for Bioscience Products & Fine Chemicals, Ajinomoto Co., Inc., Kawasaki 210-8681, Japan; yousuke.nishio.zs4@asv.ajinomoto.com (Y.N.); yoshihiko.hara.zi7@asv.ajinomoto.com (Y.H.); 3 Ajinomoto-Genetika Research Institute, Moscow 117545, Russia; gen_nonaka@agri.ru

**Keywords:** *Pantoea ananatis*, microbial production, L-glutamate, L-cysteine, isoprene, linalool

## Abstract

*Pantoea ananatis,* a gram-negative bacterium belonging to the *Erwiniaceae* family, is a well-known phytopathogen isolated from many ecological niches and plant hosts. However, this bacterium also provides us with various beneficial characteristics, such as the growth promotion of their host plants and increased crop yield. Some isolated non-pathogenic strains are promising for the microbial production of useful substances. *P. ananatis* AJ13355 was isolated as an acidophilic bacterium and was used as an excellent host to produce L-glutamic acid under acidic conditions. The genome sequence of *P. ananatis* AJ13355 was determined, and specific genome-engineering technologies were developed. As a result, *P. ananatis* was successfully used to construct a bacterial strain that produces cysteine, a sulfur-containing amino acid that has been difficult to produce through fermentation because of complex regulation. Furthermore, by heterologous expression including plant-derived genes, construction of a strain that produces isoprenoids such as isoprene and linalool as secondary metabolites was achieved. *P. ananatis* is shown to be a useful host for the production of secondary metabolites, as well as amino acids, and is expected to be used as a platform for microbial production of bioactive substances, aromatic substances, and other high-value-added substances of plant origin in the future.

## 1. Introduction

*Pantoea ananatis* is a gram-negative, rod-shaped, aerobic, or facultatively anaerobic bacteria belonging to the class *Gammaproteobacteria* and was recently reclassified into the family *Erwiniaceae* from *Enterobacteriaceae* [1,2]. *P. ananatis* was first described as *Erwinia ananas* by Serrano [3]. Some strains of *Enterobacter agglomerans*, *Erwinia herbicola*, and *Erwinia milletiae* that form part of the *E. herbicola*–*E. agglomerans* complex was assigned to the genus *Pantoea* [4]. Later, *Pantoea uredovora*, a pathogen of *Puccinia graminis*, was shown to have a high level of genomic relatedness to *P. ananas*, and the two species were synonymized [5]. *P. ananas* proposed by Mergaert et al. [5] was corrected to *P. ananatis* by Trüper and De’Clari [6]. Due to this phylogenetic history, *P. ananatis* contains various kinds of plant pathogenic strains [1]. At the same time, strains that promote plant growth that are applicable to bioremediation, or have lignocellulose degradation capacity, have been found in recent years [7].

In the field of microbial biomanufacturing, *Escherichia coli*, a model organism and a member of the family *Enterobacteriaceae*, which is a gram-negative facultative anaerobic rod, has been used industrially for the production of various substances because of its high growth rate and sugar consumption activity in the neutral pH range [8,9]. *Corynebacterium glutamicum*, a gram-positive, rod-shaped bacterium, is capable of L-glutamate fermentation [10] and has been used for the industrial production of many substances by taking advantage of its characteristic cell surface [11,12]. To date, bacterial fermentation production has been realized by mainly using *E. coli* and *C. glutamicum*, and many tools for genetic engineering have been developed for both strains [9]. However, it was clear that if both strains had the trait of robustness to pH, they would be more desirable industrial substance-producing bacteria. *P. ananatis* AJ13355 was isolated as an acidophilic bacterium [13] and has been used as an excellent host organism in previous studies to produce amino acids such as L-glutamate [14], L-cysteine [15], and isoprenoids [16,17]. The emergence of strains closely related to *E. coli* and advantageous for industrial production is important in terms of increasing the potential for substance production. The purpose of this review is to overview what is known about these *P. ananatis*, to verify their potential for the production of useful substances, and to provide perspectives on their future. We describe the potential of this strain as a new platform for bacterial bioproduction through its discovery, the development of genome engineering methods, and the production of useful metabolites based not only on academic reports but also on relevant patent information.

## 2. Characteristic of *Pantoea ananatis*

*P. ananatis* has been isolated from various environments and hosts and is well-known for its phytopathogenicity. *P. ananatis* causes disease symptoms in many economically important agronomic crops and forest tree species worldwide [1]. However, several strains have been known to improve the growth of plants [7], such as papaya [18], red pepper [19], sugarcane [20,21], poplar [22], and rice [23,24]. Kim et al. [19,25] determined the genome sequence of *P. ananatis* B1–9 isolated from the rhizosphere of the green onion and reported that enhanced red pepper crop yield by approximately three times and showed phosphate solubilization, sulfur oxidation, nitrogen fixation, and indole-3-acetic acid (IAA) production activities. *P. ananatis* AMG521, isolated as an endophyte from rice paddies, showed the capacity to synthesize siderophores, cellulose, and IAA, and the capacity to solubilize phosphate and increase rice yield [23]. *P. ananatis* strain 1.38, isolated from the rhizosphere of rice (*Oryza sativa* L.), has been reported to have phosphate solubilization activity, siderophore and auxin production, and cellulose, lipase, and pectinase activities [24]. It has long been known that *P. ananatis* produces carotenoids, and it has been reported that introducing the carotenoid biosynthesis genes of *P. ananatis* into a microorganism that does not produce carotenoids leads to the production of lycopene, astaxanthin, and β-carotene [26,27]. Ten complete genomes of *P. ananatis* have been determined and registered. Four were pathogenic and four had useful traits (Table 1).

## 3. Isolation of *P. ananatis* AJ 13355 and Genome-Editing Tools

**Figure 1:**
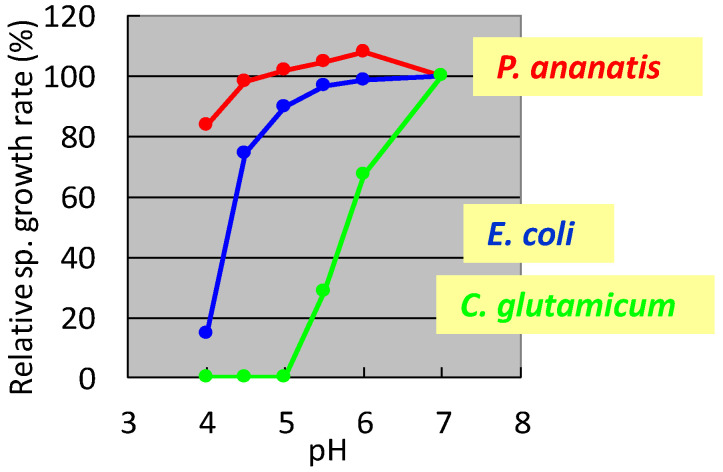
Effect of pH on the specific growth rate of *Corynebacterium glutamicum*, *Escherichia coli*, and *Pantoea ananatis*. The growth rate of each strain at pH 7 was 100%. *C. glutamicum*: green, *E. coli*; blue, *P. ananatis*: red. The data are typical results from three or more independent experiments.

In the 1990s, researchers at Ajinomoto Co., Inc. developed L-glutamate fermentation under acidic conditions. The host needed to grow at low pH and resist high L-glutamate concentrations. In the mid-1990s, specialists from Ajinomoto Co., Inc. (Kawasaki, Japan) collected a gram-negative acidophilic bacterium from the soil of a tea plantation in Iwata City (Shizuoka, Japan), which was designated as strain AJ13355. After screening various strains for these properties, *P. ananatis* strain AJ13355 was selected as the host strain for glutamate fermentation under acidic conditions. This bacterium has proven capable of growing on various sugars and organic acids at acidic and neutral pH values and is resistant to high concentrations of L-glutamic acid [35]. The effects of pH on the specific growth rates of *C. glutamicum*, *E. coli*, and *P. ananatis* are shown in Figure 1.

**Figure 2:**
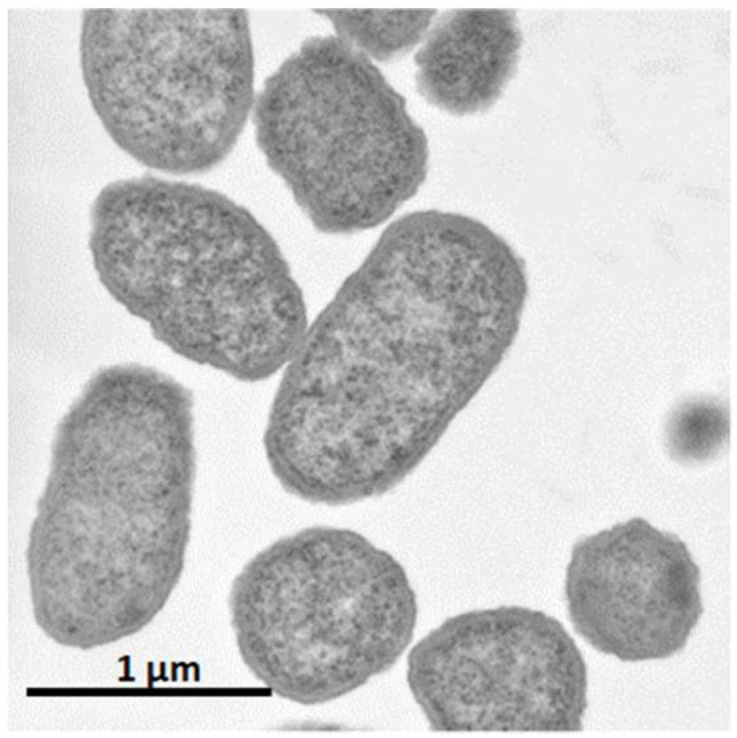
Electron micrograph of *Pantoea ananatis* AJ13355 at a magnification of ×30,200.

It was shown that *C. glutamicum* showed good growth only around neutral pH, while *P. ananatis* showed better growth than *E. coli* in acidic pH. According to standard microbiological tests on its bacteriological properties and nucleotide sequencing of its 16S rRNA [36], this strain was identified as *P. ananatis* [37]. The electron micrograph of *P. ananatis* AJ13355 shows the features of gram-negative and rod-shaped bacteria (Figure 2).

The *P. ananatis* AJ13355 strain has passed all tests required for industrialization and has been recognized as a biosafety level 1 strain. The complete genomic sequence of *P. ananatis* AJ13355 was determined and found to consist of a single circular chromosome consisting of 4,555,536 bp (DDBJ: AP012032) and a circular plasmid (pEA320) with 321,744 bp (DDBJ: AP012033). After automated annotation, 4071 protein-coding sequences were identified in the *P. ananatis* AJ13355 genome [13]. The *P. ananatis* AJ13355 genome was compared with that of *Escherichia coli*, a model organism belonging to the *Enterobacteriaceae* family to which *P. ananatis* once belonged and which was utilized in the industrial production of various substances. Short colinear regions, which are identical to the DNA sequences in the *E. coli* MG1655 chromosome, were widely dispersed along the *P. ananatis* AJ13355 genome. Conjugal gene transfer from *E. coli* to *P. ananatis*, mediated by homologous recombination between short identical sequences, has also been experimentally demonstrated [13].

To develop a general genetic tool that can be used in *P. ananatis* AJ13355, Katashkina et al. [38] constructed novel non-mobilizable derivatives of RSF1010, a well-studied broad-host-range plasmid lacking all known DNA sequences involved in the mobilization process because of the exploitation of λ Red-driven recombination between the plasmid and an in vitro-constructed linear DNA fragment. Mobilization of the obtained RSFmob plasmid was not detected in standard tests, and high stability was demonstrated in *E. coli* and *P. ananatis*, which satisfies the biosafety requirements of genetically modified organisms used in scaled-up production [38].

To develop highly active producer strains with metabolically engineered pathways, it is necessary to manipulate many genes and express them individually at different levels or under separate regulatory controls. The construction of plasmid-less marker-less strains using sequential chromosome modifications, including deletions and integration of genetic material, has many advantages for further practical exploitation of these bacteria in industry. The λ Red-recombineering technique previously developed in *E. coli* is a high-performance tool for the rapid construction of precise genome modifications, such as deletion or insertion of genetic material, nucleotide changes, modification of regulatory regions, and construction of unmarked mutations. Although the expression of λ Red genes in *P. ananatis* was highly toxic, a mutant strain, SC17(0), grew well under simultaneous expression of λ *gam*, *bet*, and *exo* genes. Using this strain, site-specific and homologous recombination of phage λ, together with the possible transfer of marked mutations by electroporation of the chromosomal DNA, were adapted to genetically engineer *P. ananatis* AJ13355 and its derivatives [39]. Minaeva et al. [40] developed a new method of foreign DNA insertion for the step-by-step construction of plasmid-less marker-less recombinant *E. coli* strains with a chromosome structure designed in advance. The dual-in/out strategy is based on the initial Red-driven insertion of artificial φ80-attB sites into the desired points of the chromosome followed by two site-specific recombination processes: first, the φ80 system is used for integration of the recombinant DNA based on selective marker-carrier conditionally replicated plasmid with φ80-attP-site; and, second, the λ system is used for excision of the inserted vector part, including the plasmid ori-replication and the marker, flanked by λ-attL/R-sites [40]. Andreeva et al. [41] adopted a dual-in/out recombineering-driven strategy to integrate DNA fragments into targeted points on the *E. coli* chromosome for application in *P. ananatis*. *P. ananatis pqqABCDEF* was cloned in vivo and integrated into the chromosomes of *P. ananatis* using the dual-in/out strategy. The introduction of a second copy of *pqqABCDEF* to *P. ananatis* SC17(0) doubled the accumulation of PQQ [41]. This new approach has facilitated the design of recombinant marker-less and plasmid-less strains. It allows for the inclusion of large artificial inserts that are difficult to introduce using commonly used PCR-based recombination procedures. Katashkina et al. [42] further improved the dual-in/out system to a method that simultaneously incorporates a few DNA fragments into specific loci on the genome. They divided the mevalonic acid pathway into acetyl-CoA to mevalonic acid (MVA), MVA to phosphomevalonate, and the remaining pathway (see Isoprenoid production). Through the improved dual-in/out system and electroporation efficiency in *P. ananatis*, they were able to handle plasmids for all three parts simultaneously. They showed that their experimental design was sufficient to remove all selection markers [42]. Consequently, it is now possible to evaluate the production of genetically engineered strains that retain the desired multiple genetic traits, resulting in an increase in breeding speed.

## 4. L-Cysteine Production

**Figure 3:**
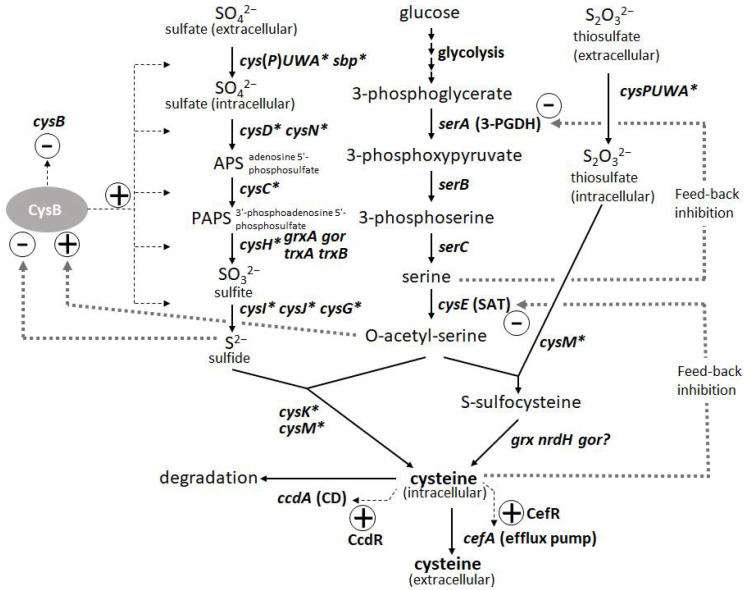
Biosynthetic pathway and regulation of L-cysteine in *Pantoea ananatis* AJ13355 [45]. Thin and thick dotted lines indicate transcription and protein regulation, respectively. Asterisk indicates CysB regulon.

L-Cysteine is an important amino acid with many applications in various industries, including pharmaceuticals, food, and cosmetics. Bacterial fermentation is well-established for many major L-amino acids in commercial production; however, L-cysteine is one of the few exceptions, for which fermentative production methods have been demonstrated only in recent years and are still under development [43,44]. Because of the cytotoxicity of L-cysteine, bacterial cells are equipped with several modes of stringent metabolic regulation that allow them to strictly control the intracellular levels of L-cysteine, making overproduction of this compound more challenging. Dissecting these multifaceted and complicated intracellular regulations and freeing them from negative feedback controls is essential to achieve efficient overproduction. These regulatory mechanisms include feedback inhibition of two key enzymes, serine acetyltransferase (SAT) and 3-phosphoglycerate dehydrogenase (3-PGDH) by L-cysteine and L-serine, respectively, along with the L-cysteine biosynthetic pathway. CysB, a master regulator of sulfur assimilation and L-cysteine metabolism, controls the expression of most of the biosynthetic and metabolic genes associated with L-cysteine at the transcriptional level (Figure 3).

The degradation of L-cysteine provides another mode of regulation wherein cysteine desulfhydrases (CDs) play a central role in protecting bacterial cells from intracellular over-accumulation. The efflux systems of L-cysteine by specific exporters add an additional layer of regulation, in which they work as a safety valve to cope with the rapid increase in its intracellular levels. Thus, L-cysteine levels are regulated by its biosynthesis, degradation, and efflux [45]. To achieve overproduction, negative regulations, namely SAT and 3-PGDH, must be deregulated, and positive regulations, namely CysB regulons of biosynthesis, must be enhanced. CDs have to be removed, but they must be combined with the enhancement of efflux transporters because the transporters contribute to recovering damaged cells by accumulating L-cysteine under deficient conditions of CDs and promoting extracellular production of L-cysteine as its oxidized form L-cystine (L-cysteine can be easily oxidized extracellularly during aerobic cultivation). Once these core factors specific to L-cysteine production are regulated, general approaches such as enhancing bottlenecks and their biosynthetic pathways and shutting down the pathways to byproducts become more effective [15]. To date, among many amino-acid-producing microbes, *P. ananatis* and *E. coli* are advanced bacterial hosts for L-cysteine production [15,46].

The introduction of mutated key biosynthetic enzymes that are free from feedback inhibition is the first step in the development of amino-acid-producing microbes. There are two key enzymes in the L-cysteine biosynthetic pathway in *P. ananatis*: SAT and 3-PGDH encoded by *cysE* and *serA*, respectively. Mutations that remove the feedback inhibition by L-cysteine have been well-studied and applied to L-cysteine production in *E. coli* (Figure 3). Effective mutations identified for 3-PGDH in *E. coli* [47] could apply to the corresponding position of this enzyme in *P. ananatis* [48]. Kai et al. [49] identified many substantial effective mutations in the SAT of *E. coli* and *P. ananatis* that abolished feedback inhibition by comparing the crystal structures of SAT with and without the allosteric inhibitor. The basic preliminary L-cysteine-producing strains can be constructed using these mutated enzymes, which typically produce traces of L-cysteine in productive media.

The second major step in genetic engineering is identifying and removing the genes involved in L-cysteine degradation. In *E. coli*, at least five CDs (TnaA, MetC, MalY, CysK, and CysM [50,51]) and one cysteine desulfidase (YhaM [52,53]) have been identified and demonstrated to exert positive effects on L-cysteine production through gene deletion. While *E. coli* has developed rather complicated degradation mechanisms involving multiple degradation enzymes, *P. ananatis* possesses the only CD encoded by *ccdA*. CcdA in *P. ananatis* is the major and strongest CD that is induced intensively by L-cysteine using CcdR encoded adjacent to *ccdA* in the corresponding locus; therefore, this CD is proposed to have a more distinctive function in L-cysteine decomposition to cope with the sudden increase in both intracellular and extracellular L-cysteine levels [54]. Deleting *ccdA* in *P. ananatis* is very effective for producing L-cysteine, as it removes detectable levels of CD activity in cells [15,45]. *P. ananatis* may have advantages over *E. coli* in managing degradation activity. *E. coli* strains with multiple gene knockouts could still exhibit significant CD activity [51], indicating that there were additional unidentified CDs that presumably negatively affected L-cysteine production. Moreover, many of the CDs in *E. coli* have significant metabolic functions (e.g., TnaA is a tryptophanase, and CysM and CysK are cysteine synthases), whose deletion may affect essential physiological functions, including growth and biosynthesis of L-cysteine.

The third step was to identify and utilize the efflux system of L-cysteine. Because of the deletion of CD gene(s), the cells exhibited poor growth that was due to the increased intracellular levels of L-cysteine. The introduction of efflux pumps increases the production of L-cysteine and recovers the damaged cells from poor growth caused by accumulated L-cysteine. YdeD and YfiK are functional efflux pumps in *E. coli* for L-cysteine [54,55]. However, since they were identified while searching for factors effective for overproducing L-cysteine, their substrates and physiological function in L-cysteine metabolism and regulation are unclear. However, *P. ananatis* has developed more specific efflux pumps of L-cysteine, associated with its metabolism and regulation. CefA and CefB of *P. ananatis* were discovered by screening for factors that confer resistance to high concentrations of L-cysteine (Figure 3). CefA was found to be inducible by L-cysteine using its counterpart regulator CefR, encoded adjacent to *cefA* in the corresponding locus; therefore, it has been proposed that it functions as a specific safety valve of L-cysteine [45]. Both CefA and CefB were demonstrated to be involved in L-cysteine overproduction in *P. ananatis*, where they significantly contributed to high-level production and recovery from L-cysteine toxicity caused by the absence of its decomposer *ccdA* [15]. *P. ananatis* may have an advantage over *E. coli* in exporting L-cysteine because it possesses more specific and efficient efflux systems that may allow the export of L-cysteine specifically without leaking out some essential metabolites required for cell viability.

If core regulatory systems that negatively control intracellular L-cysteine levels are deregulated (i.e., 3-PGDH and SAT) or turned off (i.e., CcdA) and positive systems are turned on (i.e., CefA and CefB), bottlenecks can be enhanced and unnecessary waste pathways can be attenuated. In *P. ananatis*, many biosynthetic genes have been shown to have positive effects on the overproduction of L-cysteine, including *cysM* and *cysPUWA* [15,48]. The inactivation of adverse genes such as *ydjN* and *fliY,* which encode an importer of cystine [56,57,58,59] and *yciW*, which are involved in methionine metabolism [60], was also demonstrated to have positive effects on L-cysteine production [61,62,63]. Modulation of constitutively active CysB, a master cysteine regulator that controls most of the *cys* genes, is reported to be effective in *E. coli*. This could be potentially applicable to *P. ananatis*, as this system is common in both.

Consequently, *P. ananatis* serves as an advantageous microbial host for the fermentative production of L-cysteine. It has many common features with *E. coli* in sulfur and l-cysteine metabolism. Several useful genetic tools and databases developed for *E. coli* can be applied to *P. ananatis*. Moreover, *P. ananatis* may be advantageous over *E. coli* in the modulation and management of degradation and efflux systems because it has developed native systems that are more specific and efficient for handling L-cysteine. The current best-reported demonstration of L-cysteine production by *P. ananatis* achieved 2.2 g/L levels in test tube fermentation [15], which was comparable to a recent demonstration in *E. coli* (0.6 g/L in shake flask, 5.1 g/L in fed-batch jar fermenter [64]; 1.4 g/L in shake flask, 7.5 g/L in fed-batch jar fermenter [46]). Further tuning the critical genetic elements by developing a fully optimized jar fermenter process can elevate the titer tens of times.

## 5. Isoprenoid Production

Isoprenoids are a very diverse and large group of natural products with important properties in various industries, such as pharmaceuticals, flavors, fragrances, fuels, and fuel additives; however, because they are secondary metabolites, their production in vivo is minimal despite their important physiological roles. Microbial production of many isoprenoids is desirable and has been attempted mainly in *E. coli* and *Saccharomyces cerevisiae* [65]. The biosynthesis pathway of isoprenoids is divided into two parts: formation of building blocks, isopentenyl pyrophosphate (IPP)/dimethylallyl pyrophosphate (DMAPP) of all isoprenoids, and isoprenoid synthases, which are usually derived from plants or bacteria. The biosynthesis pathways of isoprenoid precursors are categorized mainly into the mevalonic acid (MVA) and methylerythritol phosphate (MEP) pathways [66]. The theoretical yields of isoprenoids have been analyzed by using the MEP and MVA pathways and the effect of the starting molecules [67]. Yang et al. [68] reported a study of isoprene fermentation using both pathways in *E. coli*. However, many other studies have not confirmed a successful combination of both pathways. One of the reasons for this is that the metabolic behavior of the MEP pathway has not yet been fully elucidated [69]. The MVA pathway is distributed in eukaryotes and archaea, and it is known that some pathways involve different enzymatic reactions depending on the species. In some prokaryotes, the presence of the MVA pathway has been suggested by genome sequences [70]. The existence of the MVA pathway was inferred based on the genome sequence information of the *Corynebacterium variable*. Mihara et al. [71] expressed a gene corresponding to the mevalonate kinase of *C. variable* in *E. coli* and successfully measured its activity as a mevalonate kinase.

**Figure 4:**
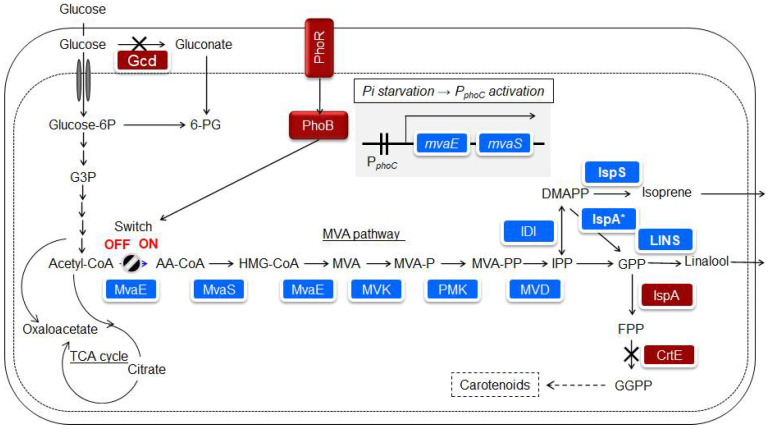
Biosynthetic pathway introduced into *Pantoea ananatis* AJ13355 to produce isoprenoids. The introduced foreign genes or enzymes are shown on a blue background. Host-derived enzymes are shown on a brown background [17].

Isoprene is mainly produced from petroleum, is widely used in chemical industries, and is one of the desired compounds for microbial production. However, isoprene can quickly evaporate in the atmosphere because of its high vapor pressure. *Bacillus subtilis* and *E. coli* are known bacterial hosts [72], and it has been reported that 1.8 g/l of isoprene was produced in *E. coli* by isopropyl-β-D-thiogalactopyranoside (IPTG) induction of a heterologous MVA pathway [73]. The IPTG, or arabinose induction system, is widely known; however, it is not a panacea in terms of the responsiveness and cost of the induction system. *P. ananatis* is also known to possess the MEP pathway and produce carotenoids. Nitta et al. [16] succeeded in developing a control system for the heterologous MVA pathway, in which isoprene production was initiated when the phosphate source in the medium was consumed by *P. ananatis*. An overview of the isoprenoid biosynthetic pathway and strain construction is shown in Figure 4 [17]. In *E. coli*, the PhoB/PhoR two-component regulatory system responded to phosphate deficiency [74]. The promoters of *phoC* and *pstS*, which were assumed to be under the control of the PhoB/PhoR two-component regulatory system of *P. ananatis*, were used for MVA production. The *mvaE* and *mvaS* from *Enterococcus faecalis*, which encode enzymes in the pathway from acetyl-CoA to MVA, are concatenated to form an artificial operon linked to the promoters of the *phoC* or *pstS* genes in *P. ananatis*. A genetically modified strain was constructed by introducing one copy of this unit into the *P. ananatis* genome. In MVA fermentation using this strain, the accumulation of MVA was low under rich phosphate source conditions in the medium, while a significant amount of MVA was observed under a limited phosphate source [16]. This result suggests that the promoters of *phoC* and *pstS* can be used to produce substances that respond to phosphate concentration. Katashkina et al. [42] studied isoprene production via the MVA pathway in *P. ananatis*.

The *mvaE* and *mvaS* genes from *E. faecalis* were introduced under the control of a phosphate responsive promoter. Three genes encoding phosphomevalonate kinase, diphosphomevalonate decarboxylase, and isopentenyl pyrophosphate isomerase from *S. cerevisiae* were artificially constructed into operons and linked to the *tac* promoter, a constitutive promoter [42]. It has been reported that eukaryotic mevalonate kinase is inhibited by phosphate compounds in the isoprenoid biosynthesis pathway. Archaea synthesize IPPs using MVA as an intermediate, but the details of the biosynthetic pathway are known to be different from those of the MVA pathway that exists widely in eukaryotes. In terms of metabolic engineering, studies are being conducted to obtain mutants that release feedback inhibition of mevalonate kinase and search for mevalonate kinases that do not have feedback inhibition [75]. Kazieva et al. [76] purified His-tagged recombinant mevalonate kinases from the methanogens *Methanosaeta concilii* and *Methanocella paludicola* using *E. coli* and characterized them for activity and feedback inhibition. These enzymes had higher catalytic efficiencies (kcat/Km) than enzymes from *Methanosarcina mazei*, which were previously reported as mevalonate kinases that were not subject to feedback inhibition. These enzymes or the enzyme from *M. mazei* were also not subjected to feedback inhibition [76]. The expression of the gene encoding mevalonate kinase from *M. paludicola* was implemented by introducing an expression unit using the tac promoter into a locus separate from the artificial operon. As a result, all genes of the MVA pathway were integrated into the genome of *P. ananatis*, and all enzymes were functional during isoprene production in *P. ananatis* [42].

Isoprene is finally produced by isoprene synthase using DMAPP as a substrate (Figure 4). Isoprene synthase has been mostly derived from plants [77]. Expression plasmids for Mucuna, Kudzu, and Populus isoprene synthases were constructed and heterologously expressed in *E. coli*. Crude protein extracts were prepared from these strains. A comparison of the isoprene production capacity per crude protein extract suggested that the enzyme from Mucuna exhibited the highest apparent activity [78]. Previous studies on poplar-derived isoprene synthase expressed in *E. coli* reported that an apparent Michaelis constant for DMAPP was 2.45 mM [79]. This suggests significant room for improvement in the use of isoprene synthase as an enzyme in material production. Nakata et al. [80] designed and evaluated isoprene synthase mutants from Mucuna based on 3D structure prediction. After conducting a simple purification, the substrate and enzyme were mixed, and the amount of isoprene generated was measured for each mutant after 18 h of incubation. A C521E mutant, which showed a 9.4-fold increase in isoprene production capacity compared to the wild-type enzyme, was successfully obtained. Isoprene synthase from Mucuna was expressed using a high-copy number vector [42]. Nitta et al. [16] analyzed the change in isoprene production over time using strains that retained these genetic traits in fed-batch cultures.

Isoprene production started when the inorganic phosphorus concentration in the culture medium was depleted, and the isoprene concentration in the off-gas of the fermenter reached 450 ppm. This indicated that the MVA pathway and isoprene synthase were successfully introduced into *P. ananatis* to synthesize isoprene [16]. In general, in the production of useful substances, a higher concentration or accumulation of the target substance has an advantage in terms of production cost. Isoprene has been reported to have an explosive range, with a lower explosive limit of 1.5 vol% and an upper explosive limit of 8.9% [81]. Because isoprene is a highly volatile substance, it requires a combination of technologies not manageable using conventional fermentation technologies, such as gas recovery technology and hazardous material handling in fermentation facilities. From the sustainability viewpoint, it is desirable to study the manufacturing method after considering the starting materials, economic rationality, and safety design.

Linalool is a monoterpenoid compound that is widely used for flavor. In the biosynthetic pathway, geranyl pyrophosphate (GPP) is a substrate of linalool synthase (Figure 4). Previous studies have shown that *E. coli* IspA produces farnesyl pyrophosphate more than GPP, and a mutant form of IspA (IspA * S80F) is effective for studying the biosynthesis of monoterpenoids [82]. Linalools also have an intramolecular chiral center; therefore, S and R forms exist. A set of strains with almost the same genetic traits as isoprene-producing strains, but with linalool synthase and IspA * instead of isoprene synthase, were constructed [83]. In addition, unlike isoprene, linalool remains in the solution and continues to adhere to the microorganisms, which may cause harmful effects such as inhibition of respiratory activity. As a result, the two-phase culture method was adopted, with isopropyl myristate added as a solvent. Thirteen candidate linalool synthases were introduced into *P. ananatis*, and their ability to produce linalool and optical activity was confirmed. The results showed that 100% S linalool synthesis was observed when the enzymes from *Malus domestica* and *Actinidia arguta* were used, and 100% R linalool synthesis was observed when the enzymes from *Ocimum basilicum* and *Streptomyces clavuligerus* were used [83]. Nitta et al. [17] heterologously expressed S-linalool synthase from *A. arguta* in *P. ananatis*, prepared a crude enzyme extract, examined the expression levels in the soluble and insoluble fractions, and found that most of the S-linalool synthase was present in the insoluble fraction. For substantial production, it is desirable to identify S-linalool synthase in the soluble fraction. The expression level in the soluble fraction was increased by adding solubilizing tags and examining the frequency of codon usage [17]. The structural features of class I terpene synthases distributed in plants, including isoprene synthase and S-linalool synthase, are relatively similar [84]. In the expression of plant-derived Class I terpene synthases in microbial hosts, from the viewpoint of enhancing the substance production capacity, it is suggested that various devices, such as the design of gene expression units, improvement of enzyme kinetics, and enhancement of solubilization rate, are necessary. Nitta et al. [17] studied the culture conditions and expression method of the enzyme for (S)-linalool production. The results showed that (S)-linalool accumulation had a production capacity of > 10 g/L. By implementing several strategies, high production capacity was achieved. The following methods were used to achieve high production capacity: (1) introduction of a tag to increase the solubility of linalool synthase, (2) increase in the ability to synthesize GPP as a precursor, which increases the activity of the enzyme in the cell, and (3) adoption of a culture method that separates the growth of the strains from the production of (S)-linalool, including a two-phase culture and phosphate-responsible promoter [17].

In an earlier study, *Streptomyces* and *E. coli* were widely used as hosts for microbial production of isoprenoids. Hoshino et al. [83] introduced genes for linalool synthase and IspA * into wild-type strains of *E. coli* and *P. ananatis*, respectively, and found that linalool accumulation was more than doubled in the wild strain of *P. ananatis*. The *P. ananatis* AJ13355 strain was also shown to be a suitable platform for producing terpene compounds. With further strain improvement and process development, the microbial production technology of monoterpenoid compounds by *P. ananatis* strain AJ13355 is expected to reach a commercial level.

## 6. Summary of *P. ananatis* Features

Among the *P. ananatis* strains identified to date, including those that promote plant growth from phytopathogens, much knowledge is accumulating on strain AJ 13355 as a strain with industrial production advantages. The characteristics of *P. ananatis* strain AJ 13355 useful for industrial production are summarized in Table 2. The pH growth range in an acidic region is broader than *E. coli* and *C. glutamicum* (Figure 1). Optimal growth temperature is 34 °C lower than *E. coli* (37 °C) and higher than *C. glutamicum* (31.5 °C) [11]. In the properties related to recombinant DNA, broad-host-range plasmids are available [38]. Genome engineering technologies have been developed to a level comparable to *E. coli* [39,40,41]. Gene regulation is generally similar to *E. coli*, but partly different, which was described in the regulation of L-cysteine [11,15]. *P. ananatis* can express actinomycetes and plant-derived genes [16,17,83], which can be advantageous for secondary metabolite production. Organic solvent tolerance is generally equivalent to *E. coli*; however, wild-strain-based production of isoprenoids was better in *P. ananatis* than in *E. coli* [83].

## 7. Conclusions

Among the isolated non-pathogenic *P. ananatis*, the *P. ananatis* strain AJ13355 was a suitable platform for producing useful substances. Genetic tools are now available for *P. ananatis* AJ13355, and it is becoming possible to breed it with the speed and accuracy of *E. coli*. It is also expected that *P. ananatis* AJ13355 has a wider acidic pH range than other common fermentation bacteria and is more resistant to stress. *P. ananatis* has different metabolisms and regulations from those of model industrial organisms, and breeding strategies can be developed to take advantage of its characteristics. It is possible to introduce heterologous biosynthetic pathways into *P. ananatis*, and it has been shown that it can be used to produce isoprenoids that are volatile and harmful to bacterial cells in two-phase cultivation processes using organic solvents.

Currently, the technology to produce value-added substances of plant origin, such as bioactive and aromatic substances, is one of the expected directions of bioproduction in the future. From the perspective of the sustainable development goals (SDGs), there is also a need for technology to produce many types of petrochemicals with plant-derived sugars produced using microbial fermentation. For this purpose, a high throughput and rapid development of a production platform using *P. ananatis* AJ13355 combined with further synthetic biology methods is required. In addition, the production system must be optimized for industrialization. We hope that these efforts will make *P. ananatis* AJ13355 a more suitable strain for the industrial production of useful substances and contribute to fermentation production.

## Figures and Tables

**Table 1:** Complete genome of *Pantoea ananatis*.

Strain	Assembly	Chromosome Size	CDS	Plasmids	Classification	Source	Reference
*Pantoea ananatis* LMG 20103	GCA_000025405.2	4,703,373	4272	0	Plant pathogen	Eucalyptus	[28]
*P. ananatis* AJ13355	GCA_000270125.2	4,555,536	4071	2	Acidophile	Soil	[13]
*P. ananatis* PA13	GCA_000233595.1	4,586,378	4131	1	Plant pathogen	Rice	[29]
*P. ananatis* LMG 5342	GCA_000283875.1	4,605,545	4675	1	N.A.	Human	[30]
*P. ananatis* R100	GCA_001543055.1	4,526,803	4353	1	Antagonist against pathogen	Rice	[31]
*P. ananatis* YJ76	GCA_002224585.2	4,671,616	4756	3	Growth-promotion	Rice	[32]
*P. ananatis* PNA 97-1R	GCA_002952035.2	4,558,720	4616	2	Plant pathogen	Onion	[33]
*P. ananatis* NN08200	GCA_004028255.1	4,743,568	4598	2	Growth-promotion	Sugarcane	[21]
*P. ananatis* LCFJ-001	GCA_016598655.1	4,499,350	N.A.	0	N.A.	*Morus alba*	N.A.
*P. ananatis* TZ39	GCA_019720835.1	4,483,976	4282	2	Plant pathogen	Rice	[34]

N.A.: not applicable.

**Table 2:** Characteristics of Pantoea ananatis AJ13355.

Classification	Characteristics	Reference
pH growth range	Broad growth range in acidic region	[13]
Optimal growth temperature	34 °C	[13]
Plasmid	Broad-host-range plasmids are available	[38]
Genome engineering	Developed to a level comparable to *Escherichia coli*	[39,40,41]
Gene regulation	Similar to *E. coli*, but partly different	[13,15]
Heterologous gene expression	Can express actinomycetes and plant-derived genes	[16,17,83]
Organic solvent tolerance	Generally equivalent to *E. coli*	[83]
